# Advancing NFL win prediction: from Pythagorean formulas to machine learning algorithms

**DOI:** 10.3389/fspor.2025.1638446

**Published:** 2025-09-12

**Authors:** Caroline Weirich, Jun Woo Kim, Youngmin Yoon, Seunghoon Jeong

**Affiliations:** ^1^ School of Global Business, Arcadia University, Glenside, PA, United States; ^2^ College of Education, University of North Texas, Denton, TX, United States; ^3^ College of Physical Education, Woosuk University, Wanju-gun, Republic of Korea

**Keywords:** NFL, neural network, Pythagorean Theorem, machine learning, sports analytics, random forest

## Abstract

This study evaluates the predictive performance of traditional and machine learning-based models in forecasting NFL team winning percentages over a 21-season dataset (2003–2023). Specifically, we compare the Pythagorean expectation formula—commonly used in sports analytics—with Random Forest regression and a feedforward Neural Network model. Using key performance indicators such as points scored, points allowed, turnovers, rushing and passing efficiency, and penalties, the machine learning models demonstrate superior predictive accuracy. The Neural Network model achieved the highest performance (MAE = 0.052, RMSE = 0.064, *R*^2^ = 0.891), followed by the Random Forest model, both of which significantly outperformed the Pythagorean method. Feature importance analysis using SHAP values identifies points scored and points allowed as the most influential predictors, supplemented by margin of victory, turnovers, and offensive efficiency metrics. These findings underscore the limitations of fixed-formula models and highlight the flexibility and robustness of data-driven approaches. The study offers practical implications for analysts, coaches, and sports management professionals seeking to optimize strategic decisions and competitive performance. Ultimately, the integration of advanced machine learning models provides a powerful tool for enhancing decision-making processes across the NFL landscape.

## 1 Introduction

American football remains one of the most popular sports in the United States, consistently holding this position since 1972. The National Football League (NFL), at the heart of this popularity, has grown into an exceptionally lucrative industry. In 2024, the combined value of the NFL's 32 teams reached approximately $190 billion, reflecting continued financial growth and robust market presence (1). Additionally, NFL viewership continues to set unprecedented records, with the 2023 playoffs averaging 38.5 million viewers, marking a notable nine-percent increase over the previous year (2).

In professional sports, success is fundamentally measured by a team's ability to win games, and NFL explicitly employs winning percentages to determine playoff eligibility and team standings. Winning percentage is traditionally calculated by dividing a team's total wins by the number of games played, with ties factored as half a win and half a loss, a standard that the NFL adopted in 1972 (3). Historically, one prominent method of predicting team success has been the Pythagorean Theorem Win/Loss formula, initially developed by Bill James for Major League Baseball. James’ formula calculates expected winning percentage based on runs scored and allowed, demonstrating impressive accuracy with a typical margin of error around 2% per team (4). Adaptations of this formula have been explored in various sports. Former Houston Rockets General Manager Daryl Morey refined the formula specifically for NFL contexts, identifying 2.37 as the optimal exponent for predicting NFL winning percentages (4). The adapted Pythagorean formula for the NFL is mathematically expressed in Equation 1:

(1) $$\mathrm{Winning}\;\mathrm{Percentage}=\frac{{\left(\mathrm{points}\;\mathrm{for}\right)}^{2.37}}{{\left(\mathrm{points}\;\mathrm{for}\right)}^{2.37}+(\mathrm{points}\;\mathrm{against}{)}^{2.37}}$$

Beyond its NFL adaptation, Pythagorean expectation formulas have been further modified for other sports, underscoring their versatility and wide applicability. For instance, research by Morey demonstrated that an exponent of 13.91 optimally predicts winning percentages in NBA contexts (4), while Caro and Machtmes (5) validated a simpler squared exponent formula to forecast win rates in college football. Further customization is evident in Davenport's logarithmic method, which adjusts exponents dynamically based on team-specific scoring data across an entire season (6). While powerful, the fixed mathematical structure of these traditional formulas inherently restricts their capacity to fully account for the nuanced, complex relationships present in competitive sports outcomes.

Recent trends in sports analytics highlight the growing potential of machine learning techniques as more flexible and robust predictive tools compared to fixed-formula methods (7). Algorithms such as random forest regression and neural networks—two prominent supervised machine learning techniques frequently applied in sports analytics—can efficiently model complex, nonlinear relationships among performance metrics (8). Unlike traditional prediction methods, these algorithms learn from historical data, capturing patterns involving offensive and defensive efficiency, schedule difficulty, margin of victory, and other influential variables. Random forest regression is valued for its interpretability and reliable accuracy in modeling intricate sports outcomes (9), while neural networks have been highlighted for their flexibility and success in capturing deeper, non-linear interactions between predictors (10).

Building upon this foundation, the current study leverages comprehensive NFL data spanning two decades (2003–2023) to empirically compare the predictive performance of the traditional Pythagorean expectation formula against data-driven machine learning algorithms—specifically random forest regression and neural network models. By evaluating these models, this study aims to identify effective methodologies for accurately forecasting NFL team winning percentages, thereby contributing valuable insights to the broader field of sport management. Sports analysts and team management can use insights derived from these predictive methodologies to optimize strategic decisions, effectively evaluate team performance, and enhance their competitive advantage in the NFL landscape.

## 2 Data and empirical methods

### 2.1 Data collection

The dataset utilized in this study was obtained from publicly accessible information provided by pro-football-reference.com. It comprises comprehensive NFL team statistics covering the seasons from 2003 through 2023. The collected data encompass details such as total games played, games won and lost, points scored (points for), points conceded (points against), average margin of victory per season, and performance statistics such as total passing yards, passes attempted, rushing yards, turnovers, penalties committed by team, etc. Across the 20-year span, the dataset contains 672 team-season observations, providing a substantial basis for predictive analysis.

Traditionally, the Pythagorean Theorem prediction method leverages only two variables—points scored and points allowed—to predict a team's winning percentage. This study incorporates this traditional method as a baseline, comparing its predictive accuracy against machine learning approaches. Random forest and neural network models are utilized as powerful analytical frameworks to capture complex patterns in the data. The random forest model (11), a robust ensemble algorithm, simultaneously analyzes multiple predictive variables, capturing complex nonlinear relationships and interactions among features included in the model. Unlike the static parameter-based Pythagorean approach, random forest regression automatically identifies and assigns appropriate weights to relevant predictors, significantly enhancing predictive flexibility and potentially improving accuracy (9). Similarly, the neural network model leverages a multilayered structure designed to adapt and learn intricate data patterns during training. Neural network is particularly adept at managing complex nonlinear relationships inherent within NFL team performance metrics such as passing yards, rushing efficiency, turnover rate, scoring consistency, and penalty impact—variables that extend beyond the simplistic points-based approach of the Pythagorean formula. The neural network approach continuously adjusts internal parameters (i.e., weights and biases of the neurons) to optimize predictive performance, offering potential superiority in capturing subtle patterns and interactions within large, multidimensional datasets (10).

Prior to model training, rigorous data preprocessing was performed. Input features underwent standardization via the StandardScaler normalization technique from scikit-learn, which adjusts variables to a consistent scale (mean of zero, standard deviation of one), ensuring optimal convergence and performance of the random forest and neural network models (12, 13). Additionally, the year variable was incorporated using one-hot encoding to control for temporal variability and annual differences (14).

### 2.2 Model architecture

This study employs three distinct methodologies to predict NFL teams’ winning percentages: the Pythagorean expectation model, random forest regression, and neural network. Each approach offers unique strengths, enabling comprehensive comparative analyses to ascertain their relative predictive power. Random forest, introduced by Breiman (15), constructs multiple decision trees during training and outputs the average prediction, effectively mitigating overfitting and improving generalization. Specifically, each tree within the random forest is constructed using bootstrap aggregation and a randomly selected subset of features, enhancing diversity among trees and reducing variance (11, 15).

The random forest architecture employed in this study leverages predictive variables including total points scored, total points allowed, average margin of victory, passing yards, rushing yards, first downs, turnovers, and penalties. Hyperparameter tuning was systematically conducted to optimize the number of trees, maximum depth, and minimum sample splits, achieving enhanced predictive accuracy and robustness. Such ensemble models are particularly adept at capturing complex, non-linear relationships among predictors, substantially outperforming simplistic linear models or fixed formulas (9).

The feedforward neural network model was developed utilizing the TensorFlow and Keras libraries, renowned for their robustness and versatility in building deep learning models (16, 17). Neural network implemented in this study consists of multiple interconnected layers of neurons—namely input, hidden, and output layers—configured to adjust parameters. The input layer receives standardized predictors, including points scored, points conceded, passing efficiency, rushing effectiveness, turnover rates, margin of victory, penalties, and encoded annual effects. These inputs are processed through two hidden layers that employ activation functions such as Rectified Linear Units (ReLU), enabling the network to learn non-linear Evaluation Metrics patterns efficiently (17). The final output layer produces predicted winning percentages. Hyperparameters such as learning rate, number of hidden layers, neuron counts, batch size, and epochs were optimized a random-split to ensure superior model performance (12, 13).

### 2.3 Evaluation metrics

Evaluating predictive model performance accurately and rigorously is critical, where forecasting outcomes can significantly inform strategic decisions. This study adopted three standard evaluation metrics: Mean Absolute Error (MAE), Root Mean Squared Error (RMSE), and the R-squared value (*R*^2^). Each metric provides distinct insights into the predictive accuracy and effectiveness of the models employed [(18); Namasudra et al, 2023; (17)]. MAE quantifies the average magnitude of errors between the predicted and actual values, ignoring their direction. The estimating Equation 2 is presented as follows:

(2) $$\mathrm{MAE}=\frac{1}{n}\overset{n}{\underset{i=1}{\sum }}\;|y_{i}-\hat{y_{i}}|$$

where *y_i_* represents actual values, $\hat{y}$_i_ represents predicted values, and *n* is the number of observations. The strength of MAE lies in its simplicity and interpretability, providing an intuitive understanding of how much, on average, predictions deviate from actual outcomes (19). RMSE measures prediction accuracy by calculating the square root of the mean squared differences between predicted and actual outcomes. The corresponding formula is specified in Equation 3:

(3) $$\mathrm{RMSE}=\sqrt{\frac{1}{n}\overset{n}{\underset{i=1}{\sum }}\;{\left(y_{i}-\hat{y_{i}}\right)}^{2}}$$

RMSE places greater emphasis on larger errors by squaring the differences, making it sensitive to outliers and particularly useful when large errors significantly impact model utility and decision-making processes (19). The R-squared value quantifies the proportion of variance in the dependent variable explained by the independent variables. The corresponding formula is shown in Equation 4:

(4) $$R^{2}\;=1-\frac{\sum_{i=1}^{n}\;{\left(yi-\hat{y}i\right)}^{2}}{\sum_{i=1}^{n}\;{\left(yi-\hat{y}i\right)}^{2}}$$

where represents the mean of observed values. R-squared value closer to 1 indicates superior predictive performance, reflecting a higher explanatory power of the model regarding observed variance. These metrics are applied to evaluate the predictive performance of three models employed in this study: the traditional Pythagorean expectation model, random forest regression, and neural network model. Applying these evaluation metrics yields a comprehensive and nuanced understanding of model effectiveness, particularly beneficial in the multifaceted and dynamic context of NFL team performance prediction.

## 3 Results

### 3.1 Comparing predictive accuracy

To evaluate the predictive accuracy of different models in estimating a team's winning percentage, we compared the performance of the traditional Pythagorean expectation model with those of the random forest and neural network models. The results are summarized in Table 1.

**Table 1:** Comparison of predicted winning percentage.

Model	Predicted winning %	MAE (Rd)	MAE (Chron)	*Δ* MAE	RMSE (Rd)	RMSE (Chron)	*Δ* RMSE	*R*^2^ (Rd)	*R*^2^ (Chron)	*Δ R* ^2^
Pythagorean	0.434	0.066	0.059	−10.6%	0.082	0.069	−15.9%	0.816	0.811	−0.005
RF	0.439	0.061	0.063	+3.3%	0.075	0.079	+5.3%	0.857	0.833	−0.024
NN	0.493	0.052	0.058	+11.5%	0.064	0.072	+12.5%	0.891	0.862	−0.029

RF, random forest; NN, neural networks; Rd, random split; Chron, chronological split; *Δ* MAE and *Δ* RMSE are percent changes: (Chron−Rd)/Rd × 100%; *Δ R*² is the absolute point change: *R*²Chron−R²Rd.

In our random forest regression analysis, we employed an ensemble of 100 decision trees to balance predictive stability against computational cost (20). Each tree was trained on a different bootstrap sample of the data and, at every split, considered a random subset of the available features, thereby reducing variance and decorrelating the individual predictors (15). We standardized all input variables to zero mean and unit variance before training, and fixed the pseudo-random seed to guarantee full reproducibility of our results. During prediction, each of the 100 trees casts an individual estimate of the winning percentage, and the final forest prediction is simply the average of these tree-level outputs. This configuration—100 trees with default maximum depth and feature-sampling settings—proved sufficient for the error curve to converge, as additional trees yielded negligible reductions in out-of-bag error. The model's predictive accuracy was strong, with a MAE of 0.061, a RMSE of 0.075, and an *R*^2^ value of 0.857 (see Table 1). These results outperformed the traditional Pythagorean expectation method across all metrics, underscoring the value of data-driven ensemble approaches in modeling team performance. Feature importance analysis further revealed the dominant influence of total points scored and points allowed on the prediction of winning percentage. Specifically, “points for” and “points_allowed” accounted for 54% and 34% of the total importance, respectively. Other meaningful, albeit less influential, predictors included rush attempts (3%), turnovers (2%), penalties (2%), passing yards (2%), and passing attempts (2%). These results suggest that while scoring remains the most significant determinant of success, additional team statistics—particularly those related to ball control and offensive efficiency—play secondary but non-negligible roles in predicting performance.

Our multilayer perceptron (MLP), a type of feedforward neural network, comprises two hidden layers, containing 64 and 32 neurons, respectively. We incorporated dropout layers with a rate of 0.2 after each hidden layer in the neural network architecture. By randomly deactivating 20% of neurons during each training iteration, dropout disrupts potential over-reliance on specific features and encourages the model to learn more generalized patterns. This regularization technique is particularly important when working with datasets that are prone to overfitting. Among the configurations tested, the combination of an 80% training size, a batch size of 20, and 100 epochs was found to be optimal based on the performance metrics. Hyperparameters were optimized via grid search with 5-fold cross-validation on the training set, and the held-out test set was used only for final evaluation. A learning rate of 0.001 strikes an optimal balance, facilitating rapid convergence while maintaining stability. The neural network model demonstrated the best overall performance, achieving the lowest MAE of 0.052, the lowest RMSE of 0.064, and the highest R² value of 0.891. This suggests that the neural network model captured the variation in actual team winning percentages more effectively than the other models.

Under the forecasting-style chronological split, performance shifts modestly relative to the random split but the ranking remains unchanged. The neural network still leads with the lowest errors and highest fit (*Δ* MAE =  + 11.5%; *Δ* RMSE = + 12.5%; *Δ R*^2^ = −0.029), followed by random forest (*Δ* MAE = + 3.3%; *Δ* RMSE = + 5.3%; *Δ R*^2^ = −0.024). The Pythagorean baseline shows slightly lower error under chronology (*Δ* MAE = –10.6%; *Δ* RMSE = –15.9%) with essentially unchanged *Δ R*^2^ (–0.005). Despite these shifts, both machine-learning models continue to outperform the Pythagorean approach overall.

**Figure 1:**
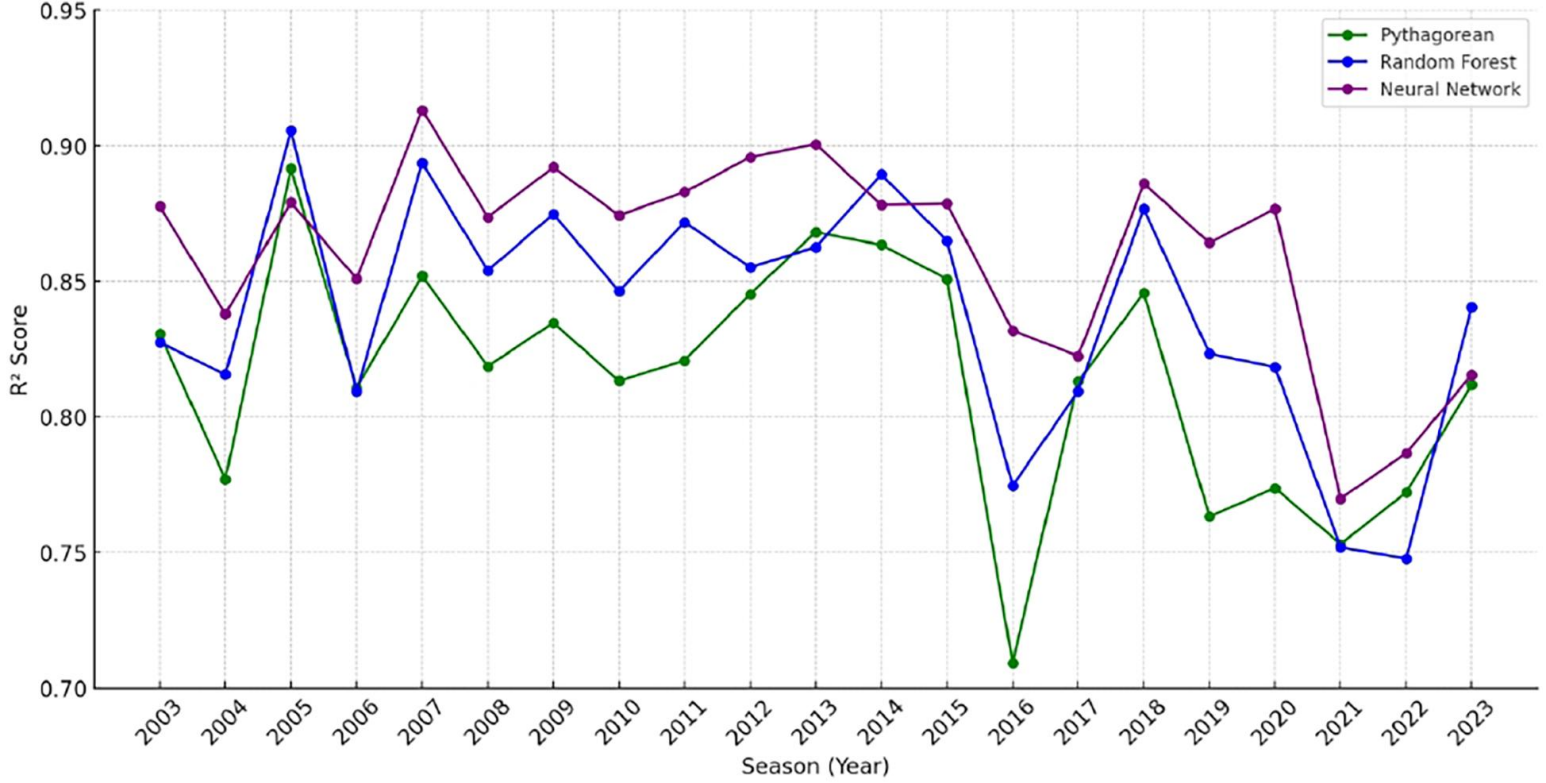
Model comparison of *R*^2^ values by season.

Additionally, the predicted average winning percentages for each model provide insight into potential under- or over-estimation tendencies. The neural network's prediction (0.493) was closest to the actual mean winning percentage (0.500), while the Pythagorean and Random Forest models predicted lower average values (0.434 and 0.439, respectively). An examination of season-by-season predictive performance reveals that the neural network model consistently produced strong results, with MAE values typically ranging between 0.05 and 0.06 and *R*^2^ values exceeding 0.80 (see Table 2). However, two notable exceptions—2016 and 2020—stand out due to elevated error metrics. In both years, the MAE exceeded 0.07, and the RMSE surpassed 0.09, indicating decreased model accuracy during these periods. Figure 1 visualizes the season-by-season R² scores of the three models from 2003 to 2023, highlighting relative consistency in neural network performance and the notable dips in 2016, 2020, and 2022.

**Table 2:** Neural network prediction results by year.

Season	Actual winning %	Predicted winning %	MAE	RMSE	*R* ^2^
2003	0.500	0.502	0.065	0.078	0.827
2004	0.500	0.508	0.067	0.081	0.816
2005	0.500	0.500	0.054	0.064	0.906
2006	0.500	0.493	0.064	0.078	0.809
2007	0.500	0.490	0.056	0.067	0.894
2008	0.500	0.490	0.066	0.078	0.854
2009	0.500	0.495	0.055	0.070	0.875
2010	0.500	0.487	0.062	0.072	0.846
2011	0.500	0.496	0.063	0.072	0.872
2012	0.500	0.488	0.058	0.073	0.855
2013	0.500	0.489	0.059	0.071	0.863
2014	0.500	0.498	0.053	0.065	0.889
2015	0.500	0.492	0.055	0.069	0.865
2016	0.500	0.503	0.078	0.094	0.775
2017	0.500	0.494	0.069	0.086	0.810
2018	0.500	0.486	0.051	0.062	0.877
2019	0.500	0.488	0.061	0.082	0.823
2020	0.500	0.488	0.071	0.091	0.818
2021	0.500	0.483	0.066	0.083	0.752
2022	0.501	0.488	0.070	0.091	0.748
2023	0.500	0.490	0.055	0.064	0.840

All numbers represent averages across 32 teams per season.

The 2016 season, in particular, was widely regarded as one of the most unpredictable in NFL history. Numerous teams significantly underperformed relative to expectations, including the Cleveland Browns, San Francisco 49ers, New York Jets, Chicago Bears, and Jacksonville Jaguars. These franchises had been expected to show signs of improvement following roster changes but instead regressed dramatically. The Browns, for instance, finished the season with just one win, down from three the previous year, despite offseason acquisitions. The Jets dropped from ten wins in 2015 to just five in 2016. Additional contributing factors to the volatility of that season include inconsistent officiating and an unusually high number of penalties, especially concerning celebration rules that were later relaxed in 2017. Furthermore, injuries to key players such as Derek Carr, Marcus Mariota, Adrian Peterson, and Rob Gronkowski disrupted team dynamics and may have reduced the predictive reliability of input metrics. The 2020 season may have been similarly impacted by disruptions related to the COVID-19 pandemic, which affected player availability, game schedules, and team performance consistency. The 2022 NFL season presented a unique set of challenges that contributed to decreased predictive accuracy in our models. While the 2016 and 2020 seasons were marked by significant unpredictability due to factors like team underperformance and the COVID-19 pandemic, the 2022 season's complexity stemmed from a confluence of unexpected team performances, significant injuries, and coaching transitions. The Tampa Bay Buccaneers and Green Bay Packers, both considered strong Super Bowl contenders, experienced offensive struggles that deviated sharply from projections. The Buccaneers, for instance, suffered unexpected losses to underperforming teams like the Carolina Panthers and Pittsburgh Steelers, highlighting the volatility of team performances during the season (21). Injuries also played a pivotal role in the season’s unpredictability. Key players returning from major injuries, such as J.K. Dobbins of the Baltimore Ravens, faced setbacks that impacted team performance. The league saw a high number of players returning from ACL injuries, introducing variability in player availability (22). Collectively, these anomalies help explain the comparatively higher prediction errors in these years.

### 3.2 Paired bootstrap test: model comparison

To rigorously compare the predictive accuracy of the models, we conducted a paired bootstrap analysis with 1,000 iterations, estimating the distribution of differences in MAE and RMSE across model pairs. Table 3 presents the mean difference and 95% confidence intervals for each comparison. The paired bootstrap analysis shows that the neural network model achieves the best predictive performance, significantly outperforming the Pythagorean method in both MAE (mean difference = −0.029, 95% CI [−0.033, −0.024) and RMSE (mean difference = −0.023, 95% CI [−0.029, −0.018), and significantly outperforming the random forest model in RMSE (mean difference = −0.014, 95% CI [−0.022, −0.006). There is no significant difference between the neural network and random forest in MAE. These findings support the neural network as the most effective predictive model for estimating a team's winning percentage.

**Table 3:** Paired bootstrap test.

Metric	Comparison	Mean difference	95% CI lower	95% CI upper
MAE	NN vs. RF	0.001	−0.005	0.005
MAE	NN vs. PY	−0.029	−0.033	−0.024
MAE	RF vs. PY	−0.029	−0.035	−0.023
RMSE	NN vs. RF	−0.014	−0.022	−0.006
RMSE	NN vs. PY	−0.023	−0.029	−0.018
RMSE	RF vs. PY	−0.009	−0.016	−0.003

NN, neural network; RF, random forest; PY, Pythagorean expectation. Negative mean differences indicate that the first model in the comparison achieved lower error than the second.

Confidence intervals (CI) were calculated using 1,000 paired bootstrap iterations.

### 3.3 Understanding feature impact through SHAP

**Figure 2:**
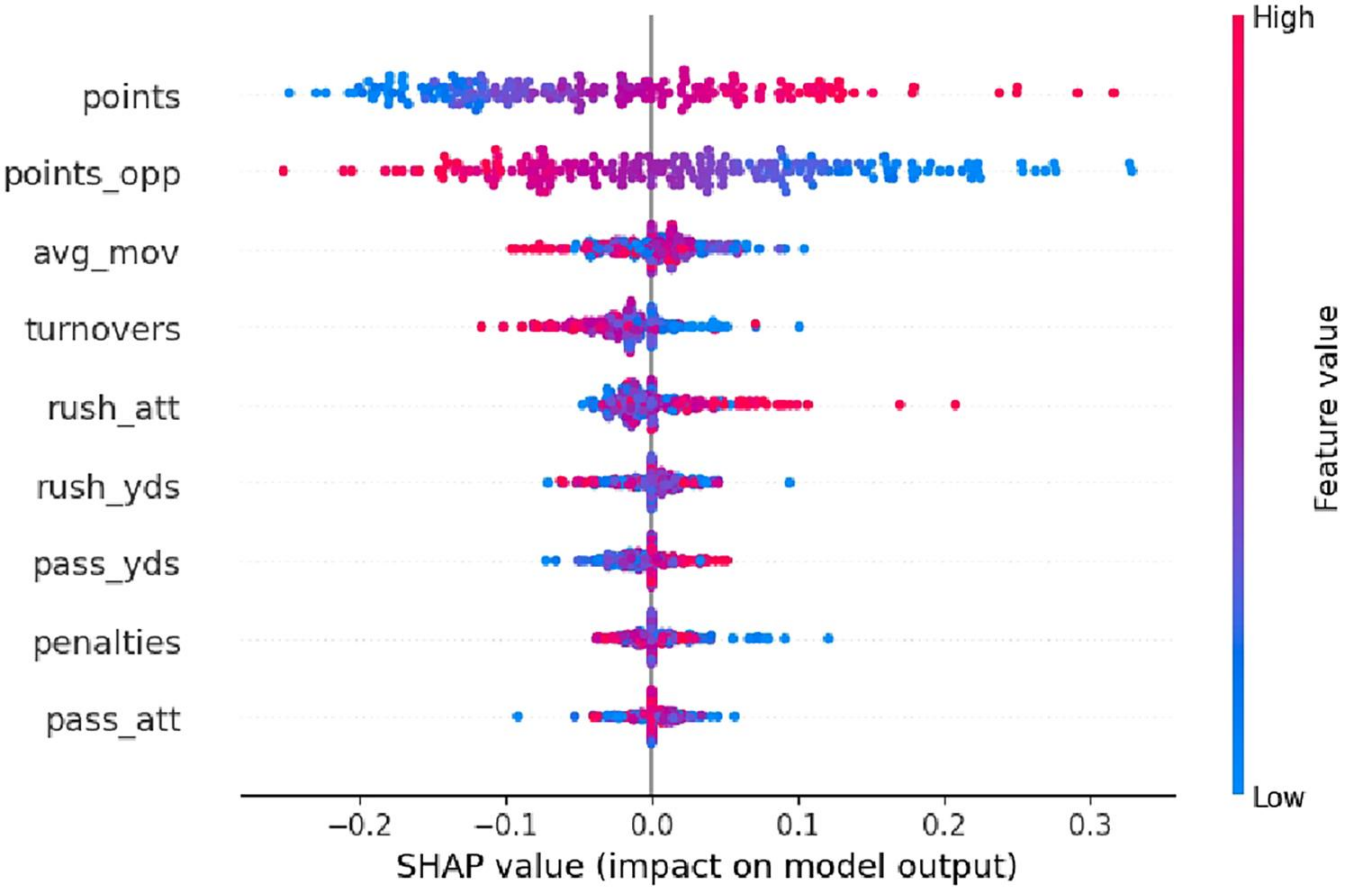
SHAP summary plot: feature impact on predicted wiin percentage (neural network model).

To better understand how various game metrics influence predicted winning percentages in the trained neural network model, we employed SHAP (SHapley Additive exPlanations) analysis. The resulting SHAP beeswarm plot visualizes the contribution of each feature to the model's output—NFL team winning percentage—across all samples in the test dataset. The features with the most significant impact on predicted winning percentages are points scored and points allowed. These two variables dominate the top of the plot with the broadest SHAP value distributions (see Figure 2). Specifically, higher point totals (shown in red) strongly increase predicted winning percentages (positive SHAP values), while lower point totals (blue) reduce them. High-scoring seasons (bright red points) almost universally exhibit large positive SHAP values, boosting predicted win rates by as much as 0.30 or more. The average margin of victory (avg_mov) and turnovers also show meaningful influence, albeit less than the core scoring variables. Higher margin values (red) generally increase predicted winning percentages, while lower or negative margins (blue) suppress predictions. Turnovers exhibit a similar trend: higher turnover counts (red) are associated with negative SHAP values, indicating that teams committing more turnovers are predicted to have lower winning percentages.

## 4 Conclusion

This study empirically evaluated the effectiveness of the traditional Pythagorean expectation formula against advanced machine learning methods, specifically random forest regression and neural network models, in predicting NFL teams’ winning percentages over a substantial 21-season dataset (2003–2023). The findings demonstrate that the machine learning models significantly outperform the traditional Pythagorean expectation approach, achieving greater predictive accuracy as evidenced by lower MAE, RMSE, and higher *R*^2^ values. Specifically, the neural network model exhibited the strongest predictive performance, with the lowest MAE (0.052), lowest RMSE (0.064), and highest *R*^2^ value (0.891). The random forest model also consistently outperformed the Pythagorean approach, indicating the advantage of leveraging data-driven ensemble methods for capturing complex nonlinear relationships among NFL performance metrics. Importantly, under the forecasting-style chronological evaluation, the neural network achieved an average MAE of 0.058. Because our outcome variable is winning percentage, it is useful to translate this value into season outcomes. In a 17-game NFL season, one game corresponds to approximately 1 ÷ 17 = 0.059 (≈5.9%) of winning percentage. Thus, an error of 0.058 equates to about one game difference in the standings. This level of predictive accuracy is practically meaningful, as a single win can determine playoff qualification, alter betting market expectations, and influence front-office or coaching evaluations.

The feature importance analysis using SHAP values further revealed critical insights into key variables influencing winning predictions. Consistent with prior literature (4, 9), points scored and points allowed emerged as dominant predictors. However, additional metrics such as average margin of victory, turnovers, rushing yards, passing efficiency, and penalties also significantly contributed to predictive accuracy, suggesting the importance of adopting comprehensive analytical frameworks rather than simplified scoring-based predictions alone.

This study contributes to existing sport management and analytics literature by validating advanced analytical methods within NFL contexts, demonstrating their accuracy and flexibility in predictive tasks compared to traditional formulas. These findings align with previous research highlighting the effectiveness of machine learning techniques in sports prediction (8, 10, 23), thereby reinforcing the growing scholarly consensus regarding their value. Specifically, previous machine learning studies in sports prediction— particularly in the NFL context (24), have typically focused on classification problems, where the outcome is categorical (i.e., win or loss). Only a limited number of studies have addressed continuous prediction tasks, such as spread and scoreline (8). In terms of predictive accuracy, classification models in the NFL context have achieved between 75% and 86%, while models predicting continuous outcomes have attained accuracy levels between 72% and 77% (8). The current study explains 89% of the variance in team winning percentage, with an average prediction error of approximately 5%, indicating a relatively higher level of predictive accuracy.

From a practical perspective, this research provides valuable implications for sports analysts, coaches, and management in professional football. Given the neural network's minimal error margin, sports analysts can utilize this approach to predict team winning percentages and playoff outcomes. Similarly, sports bettors could leverage these predictive insights to estimate team success and strategically inform betting decisions, including predicting playoff appearances and championship outcomes. NFL teams could adopt neural network-based deep learning models to evaluate and predict their performance, determining whether team performance aligns with, surpasses, or falls short of expectations (5). Additionally, such analytical tools can assist coaches and management in systematically reviewing critical in-game decisions related to scoring opportunities, fourth down strategies, turnover management, and clock management, ultimately enhancing strategic decision-making and competitive performance (5). Overall, this study underscores the substantial potential of machine learning methods, notably neural networks and random forest models, as robust decision-support tools in contemporary sport management, enhancing strategic planning and decision-making processes within professional sports organizations.

## Data Availability

Publicly available datasets were analyzed in this study. This data can be found here: https://www.pro-football-reference.com.
